# Successful private–public funding of paediatric medicines research: lessons from the EU programme to fund research into off-patent medicines

**DOI:** 10.1007/s00431-014-2398-z

**Published:** 2014-09-23

**Authors:** L. Ruggieri, V. Giannuzzi, P. Baiardi, F. Bonifazi, E. H. Davies, C. Giaquinto, D. Bonifazi, M. Felisi, C. Chiron, R. Pressler, H. Rabe, M. J. Whitaker, A. Neubert, E. Jacqz-Aigrain, I. Eichler, M. A. Turner, A. Ceci, on behalf of the GRiP Consortium

**Affiliations:** 1 Fondazione per la Ricerca Farmacologica Gianni Benzi Onlus, Via Abate Eustasio 30, 70010 Valenzano, BA Italy; 2 Consorzio per Valutazioni Biologiche e Farmacologiche, Via L. Porta 14, 27100 Pavia, Italy; 3 Paediatric European Network for Treatment of AIDS, Via Giustiniani 3, 35128 Padova, Italy; 4 Department of Paediatrics, Azienda Ospedaliera di Padova (AOPD), Via Giustiniani 1, 35128 Padova, Italy; 5 Institut National de la Sante et de la Recherche Medicale, 101 Rue de Tolbiac, 75654 Paris, France; 6 Great Ormond Street Hospital for Children NHS Foundation Trust, Great Ormond Street, London, WC1N 3JH UK; 7 Brighton and Sussex University Hospitals, Brighton, UK; 8 The University of Sheffield, Barber House 387 Glossop Road, Sheffield, S10 2HQ UK; 9 Department of Paediatric and Adolescent Medicine, University Hospital Erlangen, Loschgestrasse 15, 91054 Erlangen, Germany; 10 European Network of Paediatric Research at the European Medicine Agency, 30 Churchill Place Canary Wharf, London, E14 5EU UK; 11 Department of Women’s and Children’s Health, Institute of Translational Medicine, Liverpool Women’s NHS Foundation Trust, University of Liverpool, Crown Street, Liverpool, L8 7SS UK

**Keywords:** Paediatric clinical trials, Seventh Framework Programme, Drug development, PUMA

## Abstract

The European Paediatric Regulation mandated the European Commission to fund research on off-patent medicines with demonstrated therapeutic interest for children. Responding to this mandate, five FP7 project calls were launched and 20 projects were granted. This paper aims to detail the funded projects and their preliminary results. Publicly available sources have been consulted and a descriptive analysis has been performed. Twenty Research Consortia including 246 partners in 29 European and non-European countries were created (involving 129 universities or public-funded research organisations, 51 private companies with 40 SMEs, 7 patient associations). The funded projects investigate 24 medicines, covering 10 therapeutic areas in all paediatric age groups. In response to the Paediatric Regulation and to apply for a Paediatric Use Marketing Authorisation, 15 Paediatric Investigation Plans have been granted by the EMA-Paediatric Committee, including 71 studies of whom 29 paediatric clinical trials, leading to a total of 7,300 children to be recruited in more than 380 investigational centres.

*Conclusion*: Notwithstanding the EU contribution for each study is lower than similar publicly funded projects, and also considering the complexity of paediatric research, these projects are performing high-quality research and are progressing towards the increase of new paediatric medicines on the market. Private–public partnerships have been effectively implemented, providing a good example for future collaborative actions. Since these projects cover a limited number of off-patent drugs and many unmet therapeutic needs in paediatrics remain, it is crucial foreseeing new similar initiatives in forthcoming European funding programmes.

## Introduction

In Europe, fewer than 30 % of marketed drugs include results from paediatric clinical trials and other information on paediatric use in their documentation (Summary of Product Characteristics, SPC or Product Leaflet, equivalent to US label) [3]. The lack of paediatric medicines is particularly concerning for neonates and in serious and life threatening diseases [4, 6].

The main consequence of this situation is the widespread off-label use in paediatrics, especially in the case of old drugs that have never received a paediatric authorisation. The paediatric ‘off-label use’ specifically refers to ‘all paediatric uses of a marketed drug not detailed in the SPC’ [18]. The off-label paediatric use in Europe accounts for 45–60 % of the total number of prescriptions with rates of up to 90 % in the premature and term neonates, infants and paediatric patients admitted to intensive care units [11].

Moreover, it is well known that studies and trials involving children are affected by many methodological [1] and ethical concerns [17] as well as by economic barriers [13] resulting in difficulties to perform high-quality paediatric studies [5, 22], compliant with the existing guidelines and recommendations for high-quality paediatric studies [10, 14]. One barrier is the lack of incentives for companies to develop medicines that contain an off-patent active pharmaceutical ingredient. The entry into force of the Paediatric Regulation in 2007 (European Commission (EC) 1901/2006 as amended [8]) gave an important contribution to reduce the above-mentioned barriers and to support the development of medicines for children. Among other provisions, the Paediatric Regulation introduced a specific measure to favour work on off-patent medicines, the Paediatric Use Marketing Authorisation (the so called PUMA). This grants a 10-year period of data exclusivity in case of paediatric development of adult medicines that are not protected by a patent or supplementary protection certificate (off-patent drugs). A PUMA application should include the submission of paediatric data in accordance with an agreed Paediatric Investigation Plan (PIP) [9]. According to Article 40 of the Regulation, the European Research Framework Programmes should reserve funds to support PUMAs in case of off-patent drugs recognised as of high therapeutic interest for children and included in a ‘priority list’ (PL) adopted, on annual basis, by the European Medicines Agency (EMA) through its Paediatric Committee. In the last 6 years, such EC funds have been delivered through the Seventh Framework Programme for Research (FP7-FRP). In particular with reference to HEALTH-(2007–2013) Programme area, five calls for proposal have been released with reference to the topic 4.2-1 ‘to develop off-patent medicinal products for the paediatric population’.

These specific calls are characterized by the fact that they should respond both to the criteria for scientific excellence stated in the FP7 EC Programmes and meet standards for high-quality paediatric research as prescribed by the Paediatric Regulation (that is implementing paediatric studies to be conducted according to an agreed PIP). They should also stimulate research capacity and support the broader EU commitment to small-to-medium-sized enterprises (SMEs^1^).

This paper aims to describe the paediatric projects funded in the framework of the Paediatric Regulation and evaluate their capacity to improve public health by meeting the policy drivers that justified the funding for supporting research capacity, innovation from SMEs and high-quality paediatric studies that contribute to the development of medicines for children and progress towards PUMAs.

## Methodology

### Data sources

To collect information on the FP7 Paediatric funded projects (content and status), the following sources have been consulted:

- Community Research and Development Information Service (CORDIS) database, available from http://cordis.europa.eu/home_en.html
- Project websites^2^
- EC-EMA websites: Priority list of off-patent medicines, different versions developed from 2007 to 2013
- EMA: Published Paediatric Committee (PDCO) decisions on Paediatric Investigation Plans (PIPs), available from http://www.ema.europa.eu/ema/index.jsp?curl=pages/medicines/landing/pip_search.jsp&mid=WC0b01ac058001d129
- The European Clinical Trials Register, available from https://www.clinicaltrialsregister.eu/ctr-search/search
- The global database of clinical trials ClinicalTrials.gov, available from http://clinicaltrials.gov/
- Scientific publications, conference presentations and meeting reports related to the projects, where available
- EuOrphan, a database of orphan drugs designed/approved in Europe and in the USA [15]

### Information collected

- Funded projects and economic information (the total costs of the projects and the funds received by the European Commission);
- General information on the Research Consortia established in the framework of these projects;
- Information on the investigated active substance(s) or medicinal products, indication(s) and therapeutic area(s);
- Information on the specific measures and requirements included in the project:
  - Obligation to submit a Paediatric Investigation Plan;
  - Presence of studies to develop age-appropriate formulations (new preparations of the drugs) or forms of medicines (new dosages and strengths);
  - Subgroups of paediatric population;
- Trials and other studies (number, type and study characteristics).

Data were validated, and incomplete or inaccurate data have been sought, by e-mail contacts with the coordinators of the projects.

## Results

### Description of funded projects

#### Projects

According to the CORDIS database and other EC sources [7], in the period of 2006–2012, five calls have been launched in the context of the FP7 programme with reference to topic 4.2-1 ‘to develop off-patent medicinal products for the paediatric population’. Under the framework of these calls, a total of 76 proposals have been submitted and 20 have been funded with a success rate of 26.3 %. This analysis describes the 20 projects, listed in Table 1, devoted to paediatric studies to support a PUMA. The total amount awarded to these projects is 98.6 million euros (see Table 1).

**Table 1:** The 20 European-funded projects in the framework of FP7 and the related costs and funds

FP7 call and subprogramme area	Acronym and start year	Total project cost (€)	EU funds (€)
FP7-HEALTH-2007-B HEALTH.2007.4.2-1	Loulla and Philla, 2008	4,244,817	3,316,415
	NeoOpioid, 2008	2,881,489	2,299,164
	03K, 2008	6,836,663	5,228,003
	TINN, 2008	6,820,915	5,161,000
	NEuroSIS, 2009	7,383,283	5,623,414
	EPOC, 2009	2,575,591	1,997,862
HEALTH-2009-single-stage HEALTH.2009.4.2-1	NEMO, 2009	7,590,402	5,800,000
	NeoMero, 2010	7,734,006	5,900,000
	PERS, 2010	7,360,763	5,600,000
HEALTH-2010-single-stage HEALTH.2010.4.2-1	TINN2, 2011	6,540,008	5,000,000
	HIP trial, 2010	7,303,559	5,662,043
	DEEP, 2011	8,126,820	5,262,963
HEALTH-2011-single-stage HEALTH-2011.4.2-1	NEO-CIRC, 2011	7,814,643	5,999,167
	TAIN, 2011	5,595,432	4,203,282
	KIEKIDS, 2011	2,776,525	2,157,071
FP7-HEALTH-2013-INNOVATION-1 HEALTH.2013.4.2-1	CloSed, 2013	7,378,566	5,997,404
	GAPP, 2013	7,189,924	5,476,875
	METFIZZ, 2013	8,624,558	5,999,962
	LENA, 2013	7,702,256	5,999,991
	NeoVanc, 2014	7,882,015	5,993,000

#### Active substances

The 20 approved projects are investigating a total of 24 active substances, 8 of which (methotrexate, 6-mercaptopurine, ciprofloxacin, budesonide, doxorubicin, deferiprone, hydrocortisone and clonidine) have been granted a European Orphan Drug (OD) designation. In four cases (methotrexate, 6-mercaptopurine, hydrocortisone and deferiprone) the orphan conditions matches the Priority List indications (acute lymphoblastic leukaemia for the first two, adrenal insufficiency and sickle cell disease, respectively).

The substances are intended to treat a total of 22 paediatric indications in 10 therapeutic areas (Table 2). The most represented area is ‘infection’ accounting for four projects.

**Table 2:** Active substance(s), paediatric indication(s) and therapeutic area

Project	Active substance(S)	Therapeutic area	Addressed paediatric indication(s)	Addressed paediatric indication(s), as indicated in the PIP
TINN	Ciprofloxacin^a^	Infections	Treatment of infections in preterm and term newborns	–
	Fluconazole			–
TINN2	Azithromycin		Treatment of infections in preterm and term newborns	Prevention of bronchopulmonary dysplasia
NeoMero	Meropenem		Treatment of late onset sepsis in neonates and infants aged <3 months Treatment of bacterial meningitis in neonates and infants aged <3 months	Treatment of bacterial sepsis in neonates and infants below 3 months of age
NeoVanc	Vancomycin		Treatment of late onset bacterial sepsis caused by vancomycin susceptible bacteria in neonates and infants aged under 3 months	Treatment of bacterial sepsis caused by vancomycin susceptible bacteria in neonates and infants aged under 3 months
NeoOpioid	Morphine	Pain	Treatment of acute pain	Treatment of acute pain, treatment of moderate to severe prolonged pain.
	Fentanyl			Pre-medication before a painful medical procedure
GAPP	Gabapentin		Treatment of chronic pain	Treatment of chronic pain of neuropathic origin
Loulla and Philla	Methotrexate^a^	Malignant neoplasms	Treatment of acute lymphoblastic leukaemia	
	6-Mercaptopurine^a^			
03K	Cyclophosphamide		Treatment of paediatric malignancies	Treatment of paediatric malignant diseases including haematological malignancies as well as soft tissue sarcoma, neuroblastoma and retinoblastoma.
	Temozolomide			
EPOC	Doxorubicin^a^		Treatment of childhood cancer	
HIP trial	Dopamine	Cardiology	Management of hypotension in preterm newborns	Treatment of hypotension in the extremely low gestational age newborn. Treatment of hypotension in children
NEO-CIRC	Dobutamine		Treatment of systemic hypotension in infants	Treatment of neonatal circulatory failure in the first 72 h after birth.
LENA	Enalapril		Cardiac failure in children	
NEMO	Bumetanide	Neurology	Treatment of neonatal seizures in babies with hypoxic ischemic encephalopathy	
KIEKIDS	Ethosuximide		Treatment of absence and myoclonic epilepsy	
TAIN	Hydrocortisone^a^	Endocrinology	Treatment of adrenal insufficiency in neonates and infants	
METFIZZ	Metformin		Treatment of polycystic ovary syndrome	Treatment of polycystic ovary syndrome as adjunct to diet and exercise in adolescent girls to improve menstrual regularity and insulin resistance.
CloSed	Clonidine^a^	Intensive care/anaesthesiology	Sedation in intensive care	Sedation in intensive care
DEEP	Deferiprone^a^	Haematology	Treatment of chronic iron overload	Treatment of iron overload in paediatric patients affected by haemoglobinopathies requiring chronic transfusion and iron chelation.
PERS	Risperidone	Child and adolescent psychiatry	Treatment of conduct disorder Treatment of schizophrenia	Treatment of conduct disorder in children and adolescents with average IQ
NEuroSIS	Budesonide^a^	Respiratory and cardiovascular disorders	Prevention of bronchopulmonary dysplasia	Prevention of bronchopulmonary dysplasia (BPD) in preterm newborn infants.

^a^Received and Orphan Designation

### Research capacity

**Fig. 1:**
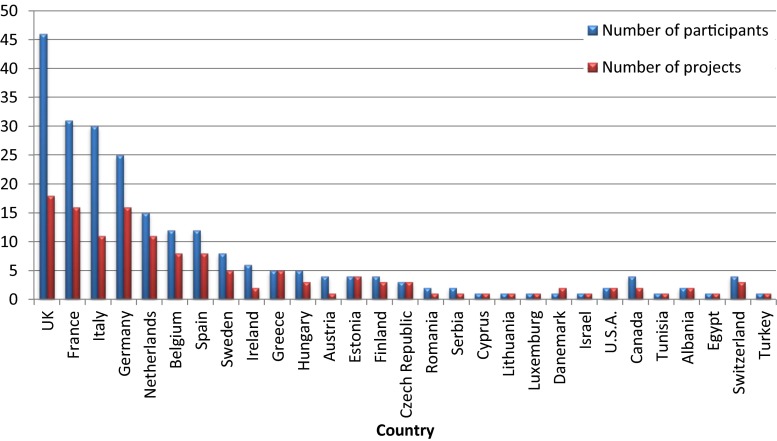
Number of participants in research consortia and number of project for each country

The 20 Research Consortia generated by the projects encompass 246 European and non-European institutions. The smallest Consortium includes four partners; the biggest one includes 18 participant members (Table 1). In addition, an average number of 6–8 investigational centres are included as third parties in each project. A total of 29 countries (22 European Member States and 7 non-European Member States) are involved with the UK, France, Italy and Germany being the most frequently represented, both in terms of number of participants and number of projects (Fig. 1). Four projects (HIP trial, DEEP, NEO-CIRC and GAPP) also include non-EU countries in their partnership. France and Italy together account for 50 % of coordinators (respectively, six and four) followed by UK and Germany (three projects each).

Most of the partners are universities and public-funded national research centres or institutions, 51 are private companies of which almost 40 are SMEs. Seven out of 20 project consortia (03K, DEEP, EPOC, TAIN, CloSed, GAPP and NEO-CIRC) include a patient association, while 9 out of 20 project consortia (NEuroSIS, EPOC, DEEP, NeoMero, PERS, LENA, GAPP, NeoVanc, CloSed) include a not-for-profit research organisation, as member or coordinator.

### Paediatric studies that contribute to the development of medicines for children

#### New forms/formulations development

Eighty percent of the projects include studies to develop new age-appropriate formulations or dosage form: ten oral new formulations (six liquid, three tablets soluble or effervescent and one granule), seven new intravenous formulations and three intravenous dosage forms.

#### Subgroups of paediatric population involved in clinical trials

**Fig. 2:**
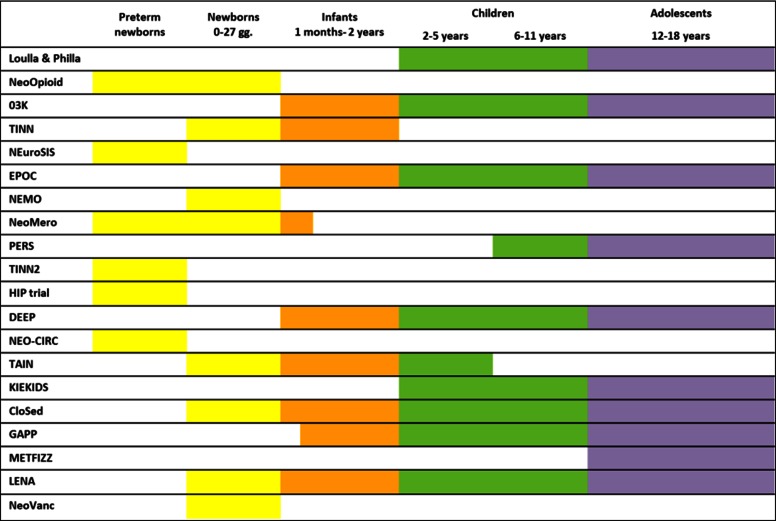
Details of paediatric age subgroups included in the clinical trials for each project

All paediatric subgroups are represented in the clinical trials (Fig. 2). In particular, 17 out of 29 paediatric clinical trials include preterm and/or term newborns. Projects NeoOpioid, NEuroSIS, NEMO, TINN and TINN2, HIP trial, NEO-CIRC and NeoVanc specifically address neonatal condition and for this reason patients to be enrolled in clinical trials are only preterm/term newborns.

#### Number and type of studies

A total of 71 studies are to be completed by the end of the 20 projects. They include studies in healthy adult volunteers (3), formulation development (20), clinical trials in children (29), non-interventional studies (6), in silico modelling (10) and non-clinical studies in animals (3).

Two out of three studies planned in healthy adult volunteers were PK, dose ranging and safety and one was a PK study. On a total of 29 paediatric trials, 16 were randomised controlled, eight on clinical pharmacology (PK/pharmacodynamics (PD)/dose finding), 9 non-randomised efficacy and/or safety and 12 were PK/PD/efficacy/safety. The estimated enrolment for all projects is 7,300 children. A pharmacogenetic sub-study is foreseen in 13 clinical trials (Table 3). Almost all trials are multicentre, involving 387 investigational sites, both in European and non-European countries. Thirteen of the paediatric trials are registered on the European Clinical Trials Database (EudraCT).

**Table 3:** Paediatric clinical trials and pharmacogenetics

Trial type	Investigational medicinal product	Study design	Pharmacogenetic
PK/PD/dose-finding	Bumetanide	–	–
	Deferiprone	–	–
	Dobutamine	–	–
	Doxorubicin	–	Analysis of polymorphisms in genes coding for the enzymes involved in the transport and metabolism
	Enalapril	–	Not available
	Fentanyl	–	Pharmacogenetic study
	Morphine	–	Pharmacogenetic study
	Fluconazole	–	Pharmacogenetic study
Efficacy and/or safety	Azithromicin	Randomised placebo-controlled	Pharmacogenetic study
	Bumetanide	Randomised placebo-controlled	–
	Dobutamine	Double-blind randomised placebo-controlled	Pharmacogenetic study
	Dopamine	Double-blind randomised placebo-controlled	Analysis of the genes coding for the transport and metabolism metabolizing enzymes to develop a pharmacogenetic assay
	Metformin	Double-blind randomised placebo-controlled	Not available
	Risperidone	Double-blind randomised placebo-controlled	–
	Risperidone	Double-blind randomised placebo-controlled	Analysis of genetic polymorphisms associated with symptoms related to risperidone use
	Metformin	Double-blind randomised placebo-controlled	–
	Meropenem	Open-label randomised active-controlled	Identification of genetic markers that may affect response to therapy
	Deferiprone	Open-label randomised active-controlled	–
PK/PD/efficacy/safety	Ciprofloxacine	Open-label non-controlled	Pharmacogenetic study
	Dobutamine	Open-label non-controlled	–
	Ethosuximide	Open-label non-controlled	–
	Hydrocortisone	Open-label non-controlled	–
	Meropenem	Open-label non-controlled	–
	Budesonide	Double-blind randomised placebo-controlled	Pharmacogenetics of steroids
	Gabapentin + morphine	Double-blind randomised placebo-controlled	–
	6-Mercaptopurine	Open-label randomised active-controlled	Pharmacogenetic analysis as primary endpoint of the CT
	Cyclophosphamide	Open-label randomised active-controlled	Not available
	Vancomycin	Randomised active-controlled	Pharmacogenetic biomarkers for treatment monitoring
	Clonidine	Double-blind randomised active-controlled	–
	Gabapentin	Double-blind randomised active-controlled	–

#### Start of the studies and patients’ enrolment

Enrolment of participants has been evaluated in 15 of 20 projects, corresponding to 23 clinical trials (5 projects, receiving approval less than a year before the survey, have been excluded from this analysis). At the time of this review (July 2014), a total of 1,400 paediatric patients (equal to 22.4 % of the estimated enrolment for all 15 projects) have been included in trials. This relates to five completed and eight ongoing trials. Ten additional trials have concluded the approval procedures and are in the process of opening.

### Support for SMEs

The partnership includes 51 private companies of which 13 are pharmaceutical companies and at least 40 meet the definition of an SME according to the information available to us. The projects are mainly coordinated by universities or other public Institutions while companies and not-for-profit research organisations are involved as coordinators in two and four projects, respectively. There are a significant number of private–public partnerships which would not have happened in the absence of pump-priming funding.

### Paediatric Investigation Plans

**Fig. 3:**
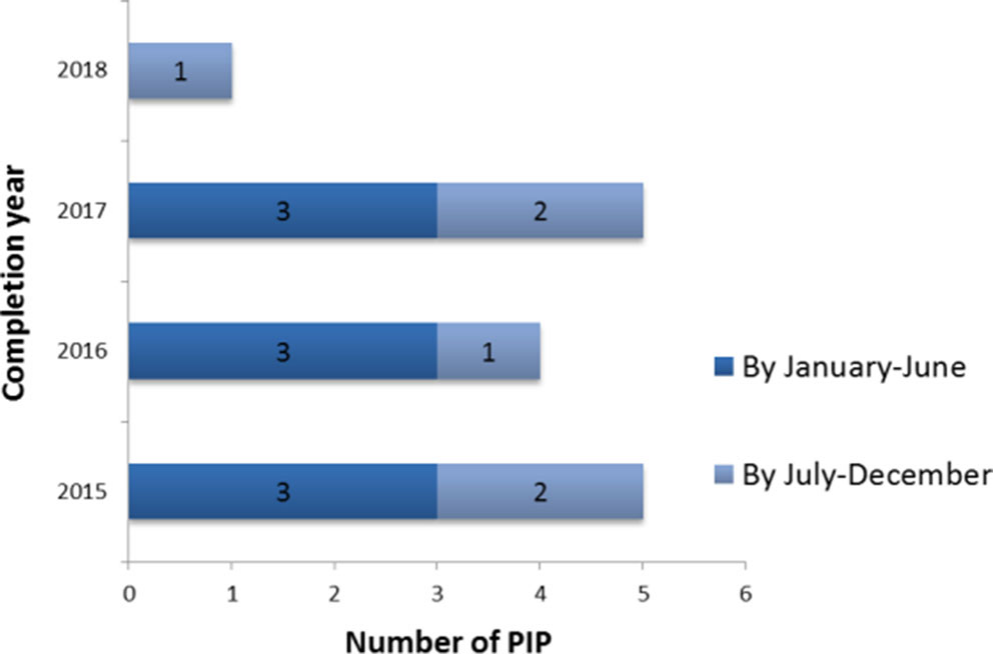
Details on the completion date of the agreed PIP

Fifteen approved PIPs referring to 14 projects are available, while two projects (TINN and LENA) have submitted a PIP application but not yet received an approved plan. As indicated in Fig. 3, no PIP has been completed so far, and the completion date of the paediatric developmental plan ranges from January 2015 to end of 2018. No deferrals are foreseen in the plans. During the PIP approval process, some changes in the projects have been performed on PDCO request. These changes have included new measures and patient populations, additional trials or studies, sample size revision, modified statistical plan, and, more frequently, additional or modified paediatric indications (Table 2). These changes were mainly prompted by valuable scientific or regulatory concerns and were accordingly integrated in the projects. However, the need to come back for approval to the EC also influenced the timing and the complexity of the projects performance. In addition, even if new measures were requested by the PDCO, no additional funds were made available by the funding authority.

### Progress towards PUMAs

PUMA applications require a PIP to be agreed, completion of all measures according to the agreed plan and confirmation of compliance with the agreed PIP. According the timelines of the FP7 projects, all of these steps should be completed within 5 years. While some projects may need extension, all of the studies will be conducted in this short period. This is different from the approach taken by many commercially funded PIPs. According to the 5-year report on the impact of the Paediatric Regulation drafted in 2012 by the EMA-PDCO [13], the number of PIPs in which studies are deferred is very high with 44 % of the approved PIPs not progressing as planned [12].

## Discussion and conclusions

Data collected and described provide evidence on how these 20 FP7 approved project are contributing to the success of the Paediatric Regulation which entered into force in 2007 and aimed to overcome the existing methodological and ethical issues affecting research in the paediatric population.

Although many of the projects are still ongoing, these results allow us to discuss the many positive achievements obtained until now. Thanks to Article 40 of the Paediatric Regulation mandate, significant funds to support drug development in children have been provided. The FP7 paediatric projects have received a total of 98.6 million euros (representing 15 % of all the EC investments for research projects related to child health in the considered time period) [7] to conduct a total of 71 paediatric studies including 32 clinical trials, corresponding to an average of only 1.4 million euros for each study or trial. This average value does not differ from similar projects funded in the FP7 [7] or other projects, such as Innovative Medicines Initiative (IMI) [16]).

However, the funding scheme of the described projects, devoted to cover the development of drugs in order to put them on the market, is a novelty for Europe considering that before FP7, regulatory clinical trials were not included in the projects funded by the commission. However, it seems that the paediatric consortia generated by the FP7 paediatric projects are conducting these studies and trials using a limited amount of money in comparison with the recognised cost of paediatric trials in an approved PIP which is estimated to be three to four times higher [2, 21]. In addition, in a very similar programme set up in the USA, the Paediatric Trials Network has received US$95 million to support 16 paediatric clinical trials, corresponding to 4.3 million euros for each trial [19]. Although it is difficult to compare the costs of conducting clinical studies in the USA and in Europe, since the payment system for investigators and researchers is different, it seems that the research consortia involved in these projects are able to manage good clinical trials with a reduced amount of resources. If this is the case, it suggests that this funding stream has unlocked a great deal of enthusiasm among child health professionals, who are also contributing to the projects with their time and other resources, while providing enough money to encourage institutions and SMEs to participate.

A very large scientific community (246 partners organisations and hundreds recruiting centres including academic, research organisations and public hospitals) covering EU and non-EU countries has been mobilised. Recent literature data demonstrated a very low involvement of researchers from academic or public research institutions in paediatric research in Europe compared with the USA [2]. The paediatric consortia born within these projects represent a critical mass of competencies that is also attracting public and private companies, scientific societies and patients’ organisations. The effect of this networking action is crucial and destined to last well beyond the end of the single research project. At present, each consortium has a very high risk to disintegrate at the end of the funding period. Thus, there is a need to sustain this capacity. The role of SMEs is striking and it is important that these and other SMEs are not discouraged from contributing to this type of work in future.

Eighty percent of the projects are developing new formulations and dosage forms of medicines specifically for the paediatric population. Age-appropriate formulations are an urgent need [20]. The 5-year report drafted in 2012 by the EMA-PDCO states that, although for authorised medicines, 26 new pharmaceutical forms were authorised for paediatric use since the entry of the Paediatric Regulation, a lack of age-appropriate formulations, in terms of safety of excipients, palatability, acceptability, dosing flexibility, accuracy and practical handling still exist [13].

The rich variety of study designs which have been adopted following peer review and detailed scrutiny by EMA suggests that high-quality studies have been tailored to the specific situations. In addition pharmacogenetic substudies intended to correlate the different pharmacological response to genetic variability are strongly represented as a fundamental step in the way to identify rational drug dosing.

The 29 ongoing paediatric trials represent a valuable percentage (14 %) of all the paediatric trials included in the EudraCT database with reference to an approved PIP, and the number of patients recruited or to be recruited (around 7,300 representing 23 % of all the paediatric patients included in clinical trials in Europe from 2007 to 2011 [13]) is highly remarkable. Furthermore, to date, 22 % of the projected enrolment for these trials is complete even though the majority of trials are yet to open. This is in contrast with the reported low recruitment capacity and difficulties with the conduct of paediatric trials [21, 22] and again demonstrates the relevance of the FP7 projects in the contest of paediatric research. The inclusion of neonates and younger paediatric subgroups is particularly important, and in contrast with the historic deficiency of medicine development in these populations. Finally, the strong engagement of commercial partners and sponsors should stimulate a critical appraisal of the PUMA concept. Commercial entities are clearly enthusiastic about developing medicines for children, but so far, only 2 PUMAs have been approved out of more than 1,000 PIPs. The PUMA is not an attractive incentive for companies [13], and in particular, SMEs, to facilitate the development and repurposing of marketed drugs for children. Developing a more appropriate incentive is of fundamental relevance for the future, since companies should be encouraged to invest in this sector. The fee reduction policy for SMEs that has been established and periodically renewed by the EMA, a larger use of free charge regulatory consultation for SME, the availability of large paediatric research infrastructures like what provided by the EnpREMA can be considered as promising instruments to increase the commitment of companies in this field.

To complete the picture provided by this analysis, some critical aspects should be discussed. As underlined in the introduction, the challenging issue for FP7 paediatric projects is to respond to different requirements imposed by the Research Programmes framework (deadline, limited resources, scientific publications, etc.) and by the Paediatric Regulation (PIP should be agreed, all the paediatric population should be covered, unmet paediatric needs prevail over scientific interest). The recommendations of the PDCO mean that relevant differences can be created from the original project mainly in term of (a) number of studies, (b) patients populations, (c) paediatric indications and (d) studies design. For these reasons, the implementation of PIPs has represented a critical point in the framework of these projects causing prolongation of the contractual procedures with the EC and often, delays in the start of the studies.

A further potential weakness is represented by time and trials management. As a result of the complexity of the administrative and ethic procedures for trial approval, each consortium has experienced the need to address specific regulatory and organisation activities that are usually outside the fields of competence of the academic and not-for-profit research groups. These activities include structured interoperability among participants, standard operating procedures to be adopted, to adhere to GCP and the requirements of trials included in the applications for MA, ethical, administrative and contractual requirements, etc.

Our results demonstrate that the majority of these challenges can be positively addressed and mainly resolved. A valid regulatory expertise seems to have been incorporated in many FP7 Paediatric Consortia that demonstrate the ability to mobilise a large scientific and clinical community. These private–public partnerships have devised clinical development plans and have conducted paediatric clinical studies that are acceptable to regulators and Ethics Committees. However, further efforts are needed, such as high level educational activities addressed to researchers and health professionals involved in paediatric trials and an increased active collaboration and resources exchange among academics, health care professionals, regulatory bodies and industry. Our expectation is that the Global Research in Paediatric (GRiP^3^), implementing an infrastructure matrix to stimulate and facilitate the development and safe use of medicines in children will provide a fundamental contribution in both these fields.

In conclusion, despite the reported difficulties and limitations, these projects are successful in many ways, are meeting the expectations of the European Commission and of the evolving Paediatric Regulation aimed to improve the health of children and led to functional paediatric drug development pathways [23]. However, much work remains to be done to make the most of the opportunities provided by these common regulatory perspectives. Therefore, new initiatives are required that will consolidate the experience and communities which have been developed by the FP7 projects. On one hand, expanded funding programmes for paediatric medicines should be made available in “Horizon 2020” or other EC Research Funds, including the Innovative Medicine Initiative (IMI) project, to meet the mandate from the European Parliament provided by Paediatric Regulation Article 40. On the other hand, it could be important that government and regulatory institutions provide the right framework allowing companies to be rewarded for their investments in paediatric clinical research.

Progress to date suggests that further work to develop medicines for children through EC-funded private–public partnerships will be productive, will provide value for money and will continue to improve public health.

## Abbreviations

CORDIS: Community Research and Development Information Service database
EC: European Commission
EMA: European Medicines Agency
EU: European
FP7-FRP: Seventh Framework Programme for Research
IMI: Innovative Medicines Initiative
Non-EU: Non-European
OD: Orphan drug
PD: Pharmacodynamics
PDCO: Paediatric Committee
PIP: Paediatric Investigation Plan
PK: Pharmacokinetics
PUMA: Paediatric Use Marketing Authorisation
SME: Small-to-medium-sized enterprise
SPC: Summary of Product Characteristics

## Footnotes

1 The category of micro, small and medium-sized enterprises (SMEs) is made up of enterprises which employ fewer than 250 persons (50 and 10 in case of small and micro, respectively) and which have an annual turnover not exceeding 50 million euros, and/or an annual balance sheet total not exceeding 43 million euros (Commission Recommendation of 6 May 2003 concerning the definition of micro, small and medium-sized enterprises, notified under document number C(2003) 1422) (2003/361/EC)

2 Not all the project websites provide the same amount of information and their update is not homogeneous

3
http://www.grip-network.org/index.php/cms/en/home
